# Transplantation and innate immunity: the lesson of natural killer cells

**DOI:** 10.1186/1824-7288-35-44

**Published:** 2009-12-30

**Authors:** Alice Bertaina, Franco Locatelli, Lorenzo Moretta

**Affiliations:** 1 Paediatric Haematology/Oncology, University of Pavia, Foundation IRCCS Policlinico San Matteo, Pavia, Italy

## Abstract

Natural killer cells have been demonstrated to play a major role in mediating an anti-leukemia effect in patients given a T-cell depleted allogeneic hematopoietic stem cell transplantation from an HLA-haploidentical family donor. In particular, donor-derived natural killer cells, which are alloreactive (i.e. KIR/HLA mismatched) towards recipient cells, significantly contribute to the eradication of leukemia blasts escaping the preparative regimen to transplantation. A recent study on high-risk pediatric acute lymphoblastic leukemia refractory to chemotherapy further highlighted the importance of donors with alloreactive natural killer cells in haploidentical hematopoietic stem cell transplantation, as it demonstrated that these cells can emerge starting from the fourth-fifth month after the allograft and persist for many months. This study represents a major breakthrough in the cure of otherwise fatal leukemias, providing information on the best criteria for choosing the optimal donor.

## 

Allogeneic haematopoietic stem cell transplantation (allo-HSCT) is largely employed for treating paediatric patients affected by many haematological conditions of both malignant and non-malignant origin, as well as by several hereditary disorders of the immune system and metabolism [1,2]. More than 40 years have elapsed since the first successful allo-HSCT, then performed using bone marrow (BM) cells [3,4], and through this procedure thousands of children have been cured of their original disease [1,2].

All this long history has taught that the success of allo-HSCT is strictly dependent on the balance between the detrimental, sometimes fatal, attack of donor immune cells against recipient tissues (i.e. graft-versus-host disease, GVHD) and the favourable reaction of donor lymphocytes towards malignant cells (i.e. graft-versus-tumor, GVT, effect) and/or pathogens capable of causing life-threatening infections during the period of profound neutropenia and lymphopenia which follows the conditioning regimen. While in the context of classical allo-HSCT from an HLA-identical sibling large part of the GVT effect is related to the attack of donor T lymphocytes against non-shared minor histocompatibility antigens expressed on tumor cells, there is clear evidence that also a selective effect of donor innate immunity (i.e. not dependent from previous encounter with the antigen) can contribute to the eradication of the malignant clone [5]. The role played by the donor innate immunity has attracted the interest of the scientific community only in recent years, when significant advances in the use of allo-HSCT have occurred. In particular, while for many years an HLA-matched sibling was the only type of donor routinely employed, in the last decade, matched unrelated volunteers and unrelated umbilical cord blood (UCB) units are being largely utilized to transplant patients lacking an HLA-identical relative [6]. The most recent, and advanced, frontier of allo-HSCT is represented by transplantation from an HLA-partially matched relative and, in this very context, the contribution of donor innate immunity to the success of the procedure has become evident. Indeed, in order to perform transplantation from an HLA-disparate donor, it is mandatory to remove from the allograft donor T cells, which, otherwise, could attack recipient-specific, either major (in large part) or minor histocompatibility, antigens, this resulting in extremely severe, often fatal, GVHD [6]. The removal of T lymphocytes, coupled with the infusion of high-doses of haematopoietic progenitors, collected from donor peripheral blood after mobilization with cytokines, has rendered possible what for many years was considered a sort of *holy grail*, namely the engraftment of donor haematopoiesis across the HLA barrier, without leading to the development of GVHD [7,8]. However, in view of the role played by donor T cells in mediating the GVT effect, it could be expected that a relevant proportion of patients given this type of allograft experience leukaemia recurrence. This expectation was only partly confirmed by the clinical results, as it became evident, mainly in adult patients affected by acute myeloid leukaemia (AML), that there was a subgroup of patients given T-cell depleted allo-HSCT from an HLA-disparate relative who had a particularly low risk of leukaemia relapse [9]. These patients belonged to the group transplanted from a donor having natural killer cells (NK) that are *alloreactive *towards recipient targets. This concept of NK-cell alloreactivity has represented a sort of revolution in the field of allo-HSCT, underlining for the first time that not only adaptive immunity, but also innate immunity is a crucial element for guaranteeing a successful patient's outcome.

NK cells are key members of the *natural *immune system, which each single individual possesses, and are of fundamental importance to limit or eradicate pathogens during the early phases of a primary infection, i.e. before T and B cells can mount efficient responses [5,10]. Indeed, NK cells and phagocytes do not require clonal expansion and can enter and defend a tissue almost as soon as it becomes infected. NK cells, derived from the haematopoietic progenitors through a common lymphoid progenitor [10], play a pivotal role in the defense against viral infections and transformed cells. The molecular mechanisms that allow NK cells to spare normal cells and kill tumor or virus-infected cells are represented by the interplay between an array of surface receptors with either activating or inhibitory function [5,10]. Indeed, NK-cell function results from the net balance between activatory and inhibitory signals (see also Figure 1). The role played by inhibitory signals represents a peculiarity of NK cells in comparison to the function of both T and B lymphocytes which is regulated only by activatory signals, and the signals delivered by inhibitory receptors present on the surface of NK lymphocytes are even more important than the activatory signals [5,10,11]. NK receptors are known as Killer Immunoglobulin-like receptors (KIR), and the inhibitory ones specifically interact with determinants that are shared by different HLA-class I molecules (referred to as KIR ligands). In a self environment, to ensure self tolerance, each individual NK cell expresses at least one inhibitory receptor specific for autologous HLA-class I and an NK-mediated attack towards autologous cells occurs only when these do not express at all or express low amounts of HLA class-I molecules.

**Figure 1:**
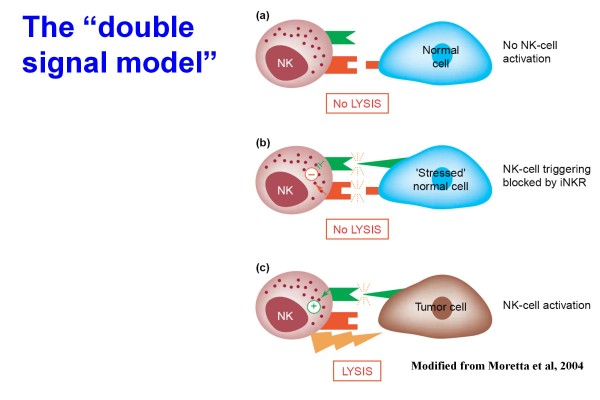
**Schematic representation of the main interactions occurring between normal natural killer (NK) cells (expressing both HLA class I-specific inhibitory receptors and activating receptors) and potential target cells**. Normal tissues deliver inhibitory signals, which block NK cells (a). Normal stressed tissues deliver simultaneously both activatory and inhibitory signals, this also resulting in block of the lytic capacity of NK cells (b). The lack of inhibitory signals, by contrast, permits NK cells to kill their targets, activatory signals playing a facilitating role (c). Modified from Moretta et al. Immunology Today 2004.

In an allogeneic setting, NK cells can kill non-self cells and the molecular basis for NK alloreactivity also involves NK-cell KIR inhibitory receptors that are specific for determinants that are shared by different KIR ligands (i.e. HLA-class I alleles). In detail, KIR2DL1 recognizes HLA-C alleles characterized by Lys at position 80 (HLA-C^Lys80^, also named C2 group), KIR2DL2/3 recognize HLA-C alleles characterized by Asn at position 80 (HLA-C^Asn80^, C1 group), KIR3DL1 recognizes HLA-B alleles sharing the Bw4 supertypic specificity (HLA-B^Bw4^, Bw4 group) [12]. This kind of inhibitory KIR receptor repertoire can lead to alloreactivity in haplo-HSCT through the mechanism of "missing self" recognition [13], provided that the donor: i) expresses a KIR-ligand which is missing in the recipient HLA genotype; and ii) expresses the specific KIR, leading to a KIR/KIR-ligand mismatch in graft-versus-host (GvH) direction. In other words, NK alloreactivity can be predicted to occur when analysis of the HLA-class I genotypes reveals that a KIR ligand is expressed in the donor but is missing in the recipient (KIR ligand-mismatch).

The existence of human "alloreactive" NK cells was originally suggested two decades ago by the hybrid resistance phenomenon in which NK cells proved to be responsible for the rejection of parental bone marrow grafts in F1 hybrid mice [14]. However, the clinical relevance of the corresponding phenomenon in human beings was not recognized till the emergence of results in adults given haplo-HSCT, demonstrating the reduction in the risk of leukaemia recurrence observed when the donor showed an NK alloreactivity with respect to the recipient HLA typing [15]. This striking finding significantly changed the criteria for choosing the HSCT donor in the context of T-cell depleted allograft from an HLA-disparate relative.

One of the most intriguing clinical questions related to donor NK alloreactivity involves the observation that this peculiar GVT effect is completely separated by the occurrence of GVHD, this indicating that NK cells are able to kill leukaemia targets, while sparing normal tissues. Clearly, the selectivity of the GVT effect displayed by NK cells cannot be attributed to an attack directed against antigens with a restricted expression on tumor cells, as the ligands of NK-cell KIR inhibitory receptors are, as mentioned above, determinants of HLA-class I molecules which are expressed on all nucleated cells of the body. Thus, the preferential lysis of tumor cells by NK lymphocytes can be interpreted only in view of the role played by activatory signals. Indeed, there is large experimental evidence that killing of allogeneic cells also depends on the surface density of activating receptors on NK cells and on the expression of their ligands on target cells [16,17]. It is currently accepted that leukaemia targets express on their cell surface ligands able to efficiently engage activatory KIR receptors [18]. Support to this interpretation is provided by the observation that, in the animal model, alloreactive NK cells not only lyse leukaemia blasts, but also kill recipient dendritic cells (DC) and T lymphocytes [10], all these targets belonging to cells deriving from the haematopoietic tissue, which, evidently, has a particular capacity to render NK cells efficiently active. This biological observation translates into other two relevant clinical advantages, namely prevention of GVHD, whose occurrence is certainly facilitated by the role played by recipient DC [19], and prevention of graft rejection, which is a complication mainly due to the effect of recipient cytotoxic T lymphocytes surviving the preparative regimen (see also Figure 2 for details).

**Figure 2:**
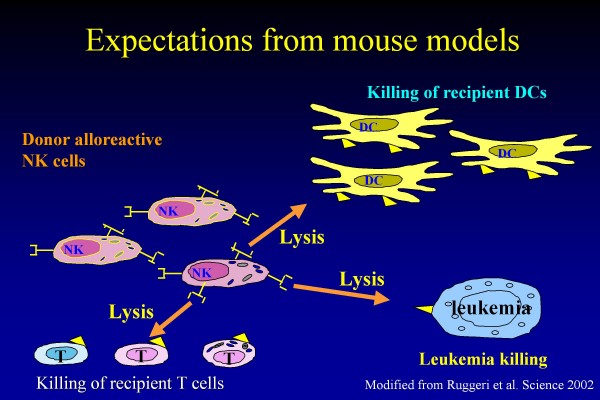
**Donor-derived alloreactive NK cells are able to kill leukaemia targets (this preventing disease recurrence), dendritic cells (DC) of the recipient (this preventing graft-versus-host disease recurrence) and cytotoxic T-lymphocytes of the recipient (this preventing graft rejection)**. Modified from Ruggeri et al. Science 2002.

If the relevance of NK alloreactivity has been demonstrated in the setting of adult myeloid leukaemia, one could argue which is the interest of this phenomenon for paediatricians. The answer comes from recent results that our groups have obtained [12]. In fact, we have demonstrated that the favourable effect mediated by donor NK alloreactivity is of crucial relevance in paediatric patients with acute lymphoblastic leukaemia (ALL), which, by far, represents the most common malignancy of childhood. In a study involving 21 children transplanted from an NK-alloreactive parent, we have documented that NK cells of donor origin able to kill leukaemia blasts emerge since the 4-5^th ^month after the allograft, persist for years and significantly contribute to the eradication of the malignant clone [12]. These results emphasize the concept that the role of innate immunity in allo-HSCT must be completely revised and re-considered also by pediatricians and, once again, demonstrated that what has been obtained in adults (namely an NK effect in AML, but not in ALL) cannot automatically be translated to children, this finding being in keeping with the old aphorism that a *child is not an adult in miniature*.

Is the mechanisms of NK alloreactivity in childhood leukaemia completely understood and the role of NK cells in transplantation fully defined? The answer is obviously no. Indeed, it remains to be addressed the unsolved problem of the need of an efficient NK alloreactive effect also in the first months after the allograft. Isolation of mature donor NK cells and their proper activation with specifically active cytokines, such as interleukin-15, could be the right solution for filling this gap and further ameliorating the clinical results of haplo-HSCT in childhood ALL. Many other biological and clinical issues also deserve critical attention for the future. Among them, the observation that, in the presence of the same kind of NK alloreactivity, mothers are better donors than fathers [20]. So far, we do not precisely know the biological explanation of this finding. What we can certainly conclude is that, also in the field of transplantation of haematopoietic progenitors, what happens during pregnancy matters.....
